# FBP1 loss contributes to BET inhibitors resistance by undermining c-Myc expression in pancreatic ductal adenocarcinoma

**DOI:** 10.1186/s13046-018-0888-y

**Published:** 2018-09-10

**Authors:** Bo Wang, Ping Fan, Jingyuan Zhao, Heyu Wu, Xin Jin, Heshui Wu

**Affiliations:** 1 Department of Pancreatic Surgery, Union Hospital, Tongji Medical College, Huazhong University of Science and Technology, Wuhan, 430022 China; 2 Department of Digestive Surgical Oncology, Union Hospital, Tongji Medical College, Huazhong University of Science and Technology, Wuhan, 430022 China; 3 Operating Room, Union Hospital, Tongji Medical College, Huazhong University of Science and Technology, Wuhan, 430022 China

**Keywords:** Fructose-1, 6-biphosphatase, C-Myc, Ubiquitination, Degradation, PDAC, BET inhibitors

## Abstract

**Background:**

Pancreatic ductal adenocarcinoma (PDAC) is one of the most lethal tumor types worldwide. BET inhibitors display anti-tumor activity in pancreatic cancer, however the cells often develop resistance after a long-term treatment and the underlying molecular basis is not fully understood.

**Methods:**

Drug screening assay in Fructose-1, 6-biphosphatase (FBP1) knockdown or overexpressing pancreatic cancer cells was performed. Tumor cell motility, FBP1 protein and mRNA changes were investigated after BET inhibitors treatment. The interaction between TRIM28 and FBP1 after BET inhibitors treatment was examined by Co-immunoprecipitation (IP) and GST pull-down. The relationship between FBP1 and c-Myc was examined by western blot, RT-qPCR and immunohistochemistry (IHC).

**Results:**

The expression of FBP1 protein increased the sensitivity of pancreatic cancer cells to JQ1. Furthermore, we showed that JQ1 stabilized FBP1 protein level by disrupting the interaction between FBP1 and TRIM28 in pancreatic cancer cells**.** Moreover, we demonstrated that FBP1 promoted c-Myc degradation through disrupting the ERK-c-Myc axis.

**Conclusions:**

FBP1 modulates the sensitivity of pancreatic cancer cells to BET inhibitors by decreasing the expression of c-Myc. These findings highlight FBP1 could be used as a therapeutic niche for patient-tailored therapies.

**Electronic supplementary material:**

The online version of this article (10.1186/s13046-018-0888-y) contains supplementary material, which is available to authorized users.

## Background

Pancreatic ductal adenocarcinoma (PDAC) is one of the most lethal tumor types worldwide and is characterized by a highly invasive and metastatic phenotype [1]. The occurrence and mortality of PDAC remain largely unchanged after decades of studies. The 5-year survival rate is still approximately 5%. Almost all chemotherapeutic agents are ineffective against PDAC [2]. Therefore, a comprehensive understanding of the pathogenesis of PDAC may unveil novel therapeutic options to improve patient diagnosis.

The bromodomain and extraterminal domain (BET) family, including BRD2, BRD3, BRD4 and BRDT, recognize acetylated histones and regulate gene transcription [3]. BET proteins usually are overexpressed in pancreatic cancer and involved in promoting tumor cell proliferation and metastasis [4]. BET inhibitors have been shown to display anti-tumor activity in KRAS-driven cancers, such as pancreatic ductal adenocarcinoma and non-small cell lung cancer [4, 5].

JQ1, one of the most studied selective inhibitors of BET proteins, has been shown the inhibition of pancreatic cancer growth in vitro and in vivo through repression of c-MYC, FOSL and HMGA2 [4, 6, 7]. However, pancreatic cancer cells often develop resistance to BET inhibitors [8]. Interestingly, it is thought that c-Myc is responsible for this resistance [9, 10]. The c-Myc protein, an oncogenic transcription factor manipulates at least 15% of genes associated with cell proliferation, differentiation and metabolism in pancreatic cancer [11]. The KRAS/ERK/c-Myc axis is the major driver of tumorigenesis in pancreatic cancer; therefore, targeting c-Myc is a promising treatment strategy for PDAC [12, 13].

PDAC is characterized by an increased aerobic glycolysis [14]. Fructose-1, 6-biphosphatase (FBP1) is a rate-limiting enzyme in gluconeogenesis that converts fructose-1, 6-bisphosphate to fructose-6-phosphate and negatively regulates aerobic glycolysis in pancreatic cancer cells [15]. The reduction of FBP1 expression in pancreatic cancer tissue compared with adjacent normal tissue is associated with a poor prognosis [16, 17]. Therefore, FBP1 functions as a tumor suppressor in pancreatic cancers. In this study, we hypothesized a novel role of FBP1 in the inhibition of tumor progression in PDAC. We demonstrated that FBP1 increased the sensitivity of pancreatic cancer to JQ1. Moreover, we showed that JQ1 stabilized FBP1 protein level in pancreatic cancer cells, and subsequently promoted c-Myc degradation through the disruption of the ERK-c-Myc axis. Collectively, our result indicated that JQ1 decreased the c-Myc expression partially via stabilizing FBP1 andthe loss of FBP1 contributed to JQ1 resistance in pancreatic cancer.

## Methods

### Cell culture

All pancreatic cancer cell lines including PANC-1 and SW1990 were purchased from the Chinese Academy of Science Cell Bank. These two cell lines were cultured in the Dulbecco’s Modified Eagle Medium (DMEM) medium (Invitrogen, USA) supplemented with 10% fetal bovine serum (FBS) (HyClone, USA). All cell lines were routinely maintained at 37 °C, 5% CO_2_ incubator.

### Plasmids, antibodies and chemicals

FBP1 wildtype (wt) and FBP1 mutant (G260R) constructs were kindly provided by Dr. Haojie Huang from the Mayo Clinic [11]. The pCMV4a-Flag-c-Myc plasmid was purchased from Addgene. FBP1 nes mutants were generated by adding a nuclear export sequence (LALKLAGLDIGS) to the FBP1 c-terminal using the KOD-Plus- Mutagenesis Kit (Toyobo, Japan). Bacterial expression vectors for GST-TRIM28 recombinant proteins were generated using the pGEX-4 T-1 backbone vector. FBP1 antibody (ab109732)was purchased from Abcam (working dilution 1:1000); beta-Tubulin (2128S) was from Cell Signaling Technology - (working dilution 1:5000); TRIM28 (ab10483) was from Abcam (working dilution 1:3000); c-Myc (5605P) was from Santa Cruz Biotechnology (working dilution 1:1000); BRD2 (ab139690) was from Abcam (working dilution 1:1000); BRD3 (A302-368A) was from Bethyl Laboratories (working dilution 1:1000); BRD4 (ab128874) was from Abcam (working dilution 1:1000). JQ1, MG132, I CBP112, puromycin and cycloheximide (CHX), were purchased from Sigma-Aldrich (Shanghai, China); MK 2206, Trametinib, GSK126, Ku55933, SB203580 and Palbociclibwere from Selleckchem (Houston, USA). Gemcitabine was obtained from Eli Lilly and Company (Indianapolis, USA). Helenalin was purchased from Cayman Chemical (Ann Arbor, USA).

### Western blot of cells and tissue specimens

The ethics of using human tissue (8 pairs of matched pancreatic cancer/adjacent noncancerous tissues) was approved by the local ethics committee (Tongji Medical College, China), and written informed consent was obtained from patients prior to surgery. The cells or the tissue specimens were lysed with lysis buffer containing 1% protease and phosphatase inhibitors as described previously [18]. The protein concentration was determined with a protein assay kit (Pierce Biotechnology, USA). Equal amounts of protein for each sample were separated using SDS-PAGE gels and transferred onto PVDF membranes (Pierce Biotechnology, USA). The membranes were subsequently blocked in 5% not-fat milk for 1 h at room temperature, followed by incubation with primary antibody overnight at 4 °C. The membranes were then washed with 1× TBST and incubated with a secondary antibody for 1 h. Finally, the membranes were treated with ECL detection reagents and exposed to X-ray films.

### Real-time RT-PCR

The total RNA was extracted from the cells using Trizol reagent (Thermo Fisher Scientific, USA). First strand cDNA was synthesized from 1 μg of RNA using a cDNA Reverse Transcription kit, and real-time PCR analysis was carried out with a PCR kit according to the manufacturer’s protocols. The two kits were purchased from Takara Bio Inc. (Shigo, Japan). All the values were normalized against actin, and the 2-ΔCt method was used to quantify fold change. Primer for RT-qPCR is provided in Additional file 1: Table S1.

### Co-immunoprecipitation (co-IP)

Cells were harvested and lysed by IP buffer (50 mM Tris-HCl, pH 7.4, 150 mM NaCl, 1% Triton X-100, 1% sodium deoxycholate and 1% protease inhibitor cocktails) on ice for more than 15 min. Cell lysate was centrifuged for 10 min at 13,000 rpm at 4 °C, and the supernatant was transferred to a new tube. The supernatant was incubated with primary antibodies and protein A/G agarose beads (Thermo Fisher Scientific, USA) with gentle rocking at 4 °C overnight. The next day, the beads were washed six times with IP buffer on ice, and then subjected to western blotting analysis.

### Glutathione S-transferase (GST) pull-down assay

Cells were lysed with cell lysis buffer (20 mM Tris-HCl (pH 7.5), 150 mM NaCl, 0.1% Nonidet P40, 1 mM DTT (dithiothreitol), 10% glycerol, 1 mM EDTA, 2.5 mM MgCl2 and 1 μg/ml leupeptin) for 30 min at 4 °C. GST fusion proteins were immobilized on glutathione-Sepharose beads (GE Healthcare Lifesciences, USA). After washing with lysis buffer, the beads were incubated with cell lysates for 4 h. The beads were then washed four times with binding buffer and re-suspended in sample buffer. The bound proteins were subjected to SDS/PAGE and western blotting analysis.

### Tissue microarray and immunohistochemistry (IHC)

The tissue microarray slides were purchased from Biomax US (lot no. PA1001a). The tissue microarray specimens were immunostained with FBP1 (Abcam, ab109732, working dilution 1:5000) and c-Myc antibodies (Santa Cruz Biotechnology, 5605P working dilution 1:100) as described previously [17, 19]. Staining intensity was scored in a blinded fashion: 1 = weak staining at 100× magnification but little or no staining at 40× magnification; 2 = medium staining at 40× magnification; 3 = strong staining at 40× magnification. The degree of immunostaining was reviewed and scored by two independent pathologists who were blinded to the clinical details. The scores were determined by the percentage of positive cells multiplied by the staining intensity.

### RNA interference

The lentivirus-based control and gene-specific shRNAs were purchased from Sigma-Aldrich. Lipofectamine 2000 was used to transfect 293 T cells with shRNA plasmids and viral packaging plasmids (pVSV-G and pEXQV). After 24 h transfection, the medium was replaced with fresh DMEM, containing 10% FBS and 1 mM of sodium Pyruvate. Next, 48 h post transfection, the virus culture medium was collected and added to PANC-1 or SW1990 cells supplemented with 12 μg/ml of polybrene. After 24 h infection, the infected cells were selected with 10 μg /ml of puromycin. The shRNA sequence information is provided in Additional file 1: Table S2.

### Cell proliferation assay

Cell viability was evaluated using the MTS assay according to the manufacturer’s instructions (Abcam, USA). Briefly, the pancreatic cancer cells (1 × 10^3^ cells) were seeded in 96-well plates with 100 μl of culture medium. The cells were treated with serial concentrations of small molecular inhibitors. After 72 h, each well of the cells was added 20 μl of MTS reagent (Abcam, USA) and incubated for 1 h at 37 °C in standard culture conditions. The absorbance was measured in a microplate reader at 490 nm.

### Glucose consumption and lactate production measurement assay

Pancreatic cancer cells (5 × 10^4^) were seeded in 6-well plates and cultured in DMEM medium without phenol red (Invitrogen, USA). After 24 h plasmid transfection or 48 h lentivirus transduction, the spent medium was collected. Glucose concentrations in the spent medium were measured using a glucose (GO) assay kit, according to the manufacturer’s instructions (Sigma-Aldrich, USA). Glucose consumption was calculated by subtracting the difference in glucose concentrations between the spent medium and the unused medium. Lactate levels were measured using a lactate assay kit (Eton Bioscience, USA).

### Generation of PDAC xenografts in mice

The BALB/c-nu mice (4–5 weeks of age, 18–20 g) were purchased from Vitalriver (Beijing, China) and randomly divided into four groups (*n* = 4/group) for subcutaneously inoculation with 5 × 10^6^ of PANC-1 cells lentivirus infected with shControl, shFBP1, shc-Myc or both shFBP1 plus shc-Myc in the left dorsal flank of mice. The tumors were examined every other day for 21 days; the length and width measurements were obtained with calipers to caculate the tumor volumes by using the eq. (L x W^2^)/2. On day 21, the animals were euthanized, and the tumors were excised and weighed. All the animal experimental procedures were approved by the Ethics Committee of Tongji Medical College, Huazhong University of Science and Technology.

### Statistical analysis

Statistical analyses were performed with one-sided or two-sided paired Student’s t-test for single comparison and one-way ANOVA with a post-hoc test for multiple comparisons. *p* value < 0.05 was considered statistically significant. All the values are expressed as the means ± SD.

## Results

### FBP1 is responsible for modulating the BET inhibitor sensitivity in PDAC

**Fig. 1:**
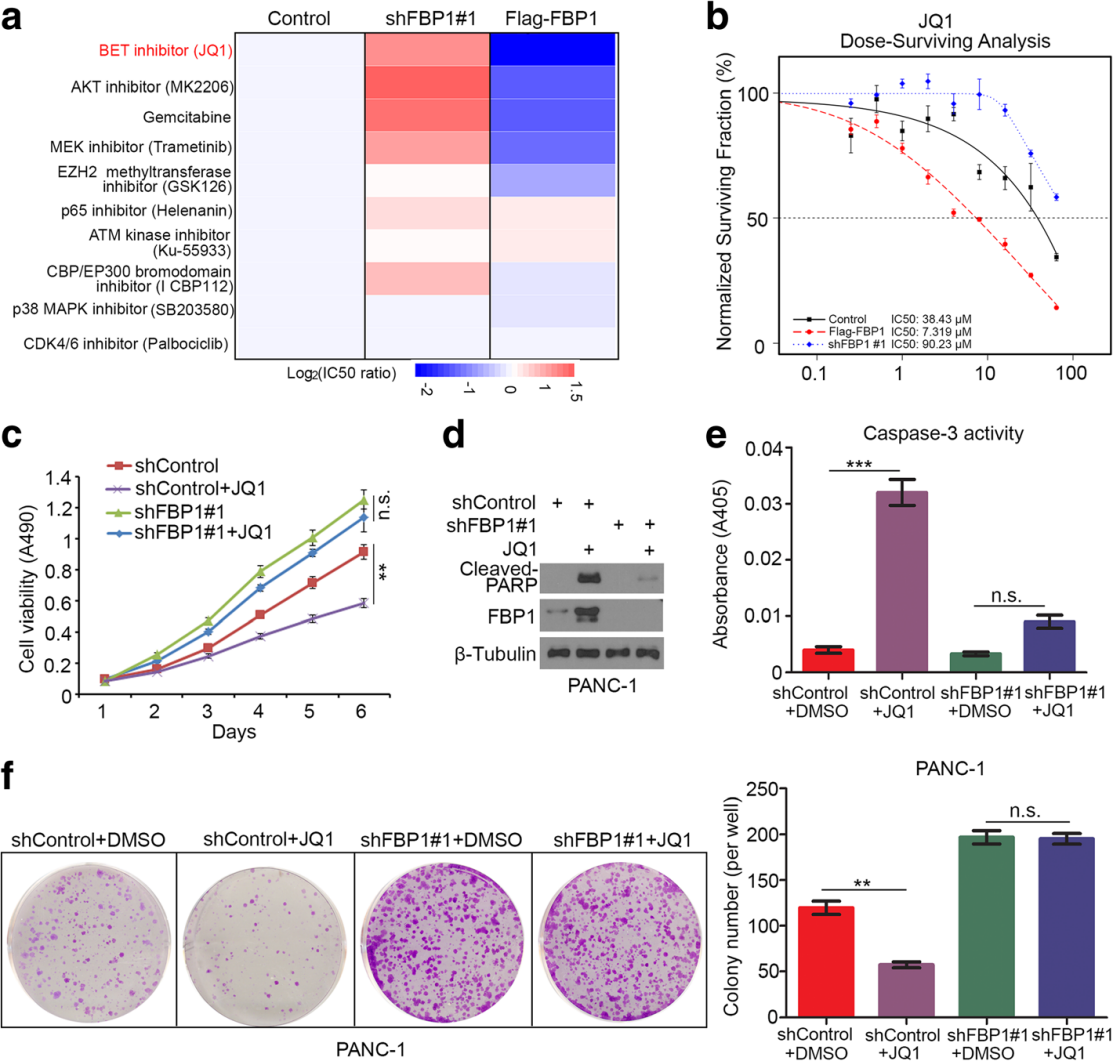
FBP1 is responsible for modulating the BET inhibitor sensitivity in PDAC. **a**, PANC-1 cells were infected with lentivirus expressing control, FBP1-specific shRNAs. After 48 h infection, shControl cells were transfected with pcDNA3.1 or Flag-FBP1 constructs. All cells were treated with different doses of indicated chemicals 24 h post-transfection. The cell viability was measured by MTS assay. Heat map showing the IC50 ratio (log_2_ (IC50 ratio)) between shControl versus shControl, knockdown FBP1 versus shcontrol or overexpression FBP1 versus control treated with indicated chemicals. **b**, PANC-1 cells were infected with lentivirus expressing control, FBP1-specific shRNAs. After 48 h infection, shControl cells were transfected with pcDNA3.1 or Flag-FBP1 constructs. Cells were treated with different doses of JQ1 24 h post-transfection. The cell viability was measured by MTS assay. Data shown are mean values ± SD from six replicates. **c-f**, PANC-1 cells were infected with lentivirus expressing control or FBP1-specific shRNAs. After 72 h infection, cells were harvested for MTS assay (**c**), western blotting (**d**), caspase 3 activity assay (**e**) and colony formation assay (**f**). All data are shown as mean values ± SD (*n* = 3). n.s., not significant, ** *p* < 0.01 comparing to the shControl group

The *FBP1* is a well-known tumor suppressor gene that exhibits low expression or loss of expression in many types of solid tumors [20–22]. Given the importance of the inhibition of cancer progression by FBP1 and the unclear underlying molecular mechanism for this, we performed a drug screening assay in FBP1 knockdown or overexpressing pancreatic cancer cells (PANC-1) and compared the IC50 values of each small molecule with that of the controls (Fig. 1a). We found that the IC50 values of JQ1, the most studied BET inhibitor [23], in FBP1 knockdown group was higher than control group (Fig. 1a and b). In contrast, the IC50 value of JQ1 in FBP1 overexpression group was lower than that of the control (Fig. 1a and b). These data suggest that FBP1 is involved in regulating JQ1 sensitivity in pancreatic cancer (Fig. 1b), using gemcitabine as a positive control (Fig. 1a), consistent with previous findings showing that FBP1 loss is responsible for gemcitabine resistance in pancreatic cancer [17]. In order to verify the role of FBP1 in sensitizing PDAC cells to JQ1-induced apoptotic death, PANC-1 cells were treated with JQ1 alone or in combination with FBP1-targeted shRNAs. The knockdown of FBP1 not only increased the pancreatic cancer cells viability (Fig. 1c and f), but also promoted PANC-1 cell resistant to JQ1 drug via decreasing the cleaved PARP expression and caspase-3 activity (Fig. 1c-1f). Together, our data indicate that FBP1 loss plays a vital role in BET inhibitors resistance in PDAC cells.

### JQ1 positively regulates FBP1 protein stability in PDAC

**Fig. 2:**
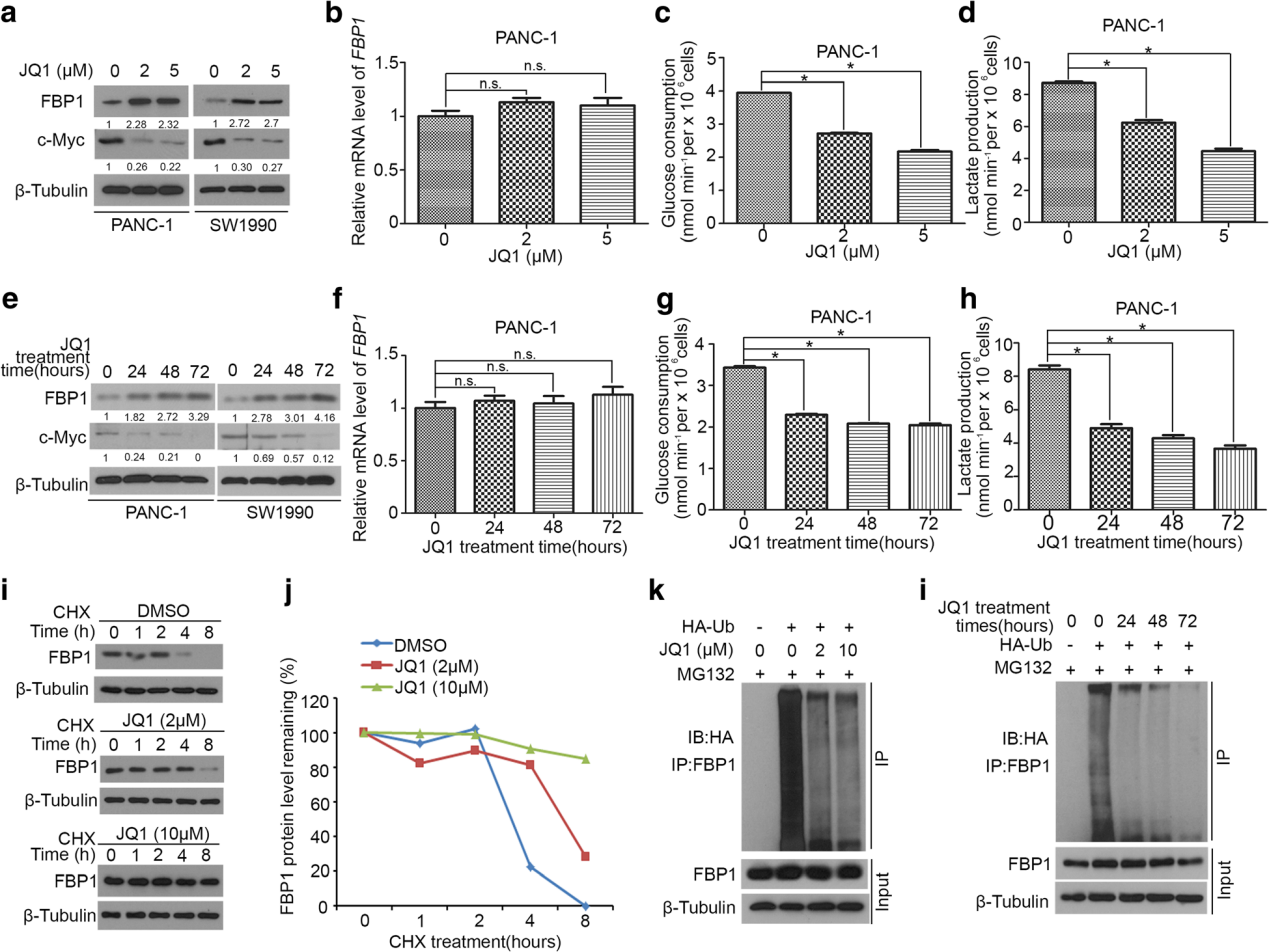
JQ1 positively regulates FBP1 protein stability in PDAC. a-d, PANC-1 and SW1990 cells were treated with different dose of JQ1 as indicated. After 24 h, cells were harvested for western blot analysis (**a**), RT-qPCR (**b**). Data are shown as means ± SD (*n* = 3). n.s., not significant. Measurement of glucose consumption (**c**) and L-lactate production (**d**) in the spent medium of PANC-1 cells treated with different dose of JQ1 as indicated, medium was collected after 24 h. Data are shown as means ± SD (*n* = 3). *, *P* < 0.05. **e-h**, PANC-1 and SW1990 cells were treated with 2 μM of JQ1. After different time points as indicated, cells were harvested for western blot analysis (**e**) and RT-qPCR (**f**). Data are shown as means ± SD (n = 3). n.s., not significant. Measurement of glucose consumption (**g**) and L-lactate production (**h**) in the spent medium of PANC-1 cells treated with 2 μM of JQ1, medium was collected in different time point as indicated after JQ1 treatment. Data are shown as means ± SD (n = 3). *, *P* < 0.05. **i** and **j**, PANC-1 cells were treated with different dose of JQ1 as indicated. After 24 h, cells were treated with 50 μg/μl cycloheximide (CHX). At different time points, cells were harvested for western blot analysis. At each time point, the intensity of FBP1 was normalized to the intensity of β-Tubulin (loading control) first and then to the value at the 0-h time point. **k**, PANC-1 cells were transfected with the indicated constructs. 24 h post transfection, cells were treated with different dose of JQ1 as indicated for another 24 h,then cells were harvested for western blot analysis. Cells were treated with or without 20 μM of MG132 for 8 h before harvested. **l**, PANC-1 cells were transfected with the indicated constructs. 24 h post-transfection, cells were treated with 2 μM of JQ1. After different time points as indicated, cells were harvested for western blot analysis. Cells were treated with or without 20 μM of MG132 for 8 h before harvested

Given that FBP1 regulates the sensitivity of JQ1 in PDAC cells, the underlying mechanism is unclear. Intriguingly, the data in Fig. 1d indicate that JQ1 increase the expression of FBP1 in PANC-1 cells. Therefore, we use different pancreatic cancer cell lines, PANC-1 and SW1990 to verify this phenomenon. We showed that JQ1 treatment increased FBP1 protein level (Fig. 2a and e) but not the mRNA level (Fig. 2b and f) in dose-and time- dependent manners in both PANC-1 and SW1990 pancreatic cancer cells. Since JQ1 mainly block the transcriptional activity of Bromodomain and Extra-Terminal motif (BET) proteins BRD2, BRD3, BRD4, and BRDT [24, 25], we knocked down of BRD2, BRD3 or BRD4 respectively with corresponding specific shRNAs in PANC-1 cells (Additional file 1: Figure S1a, c and 1e) and found that neither knockdown of BRD2, BRD3 nor BRD4 changed the expression of FBP1 in PANC-1 cells (Additional file 1: Figure. S1a-f). Furthermore, due to FBP1 acting as a key enzyme in gluconeogenesis that antagonizes “Warburg effect”, we found that JQ1 treatment prevented the glucose consumption and lactate production in PANC-1 cells (Fig. 2c, d, g and h), which was consistent with the alteration of FBP1.

Since JQ1 increased FBP1 protein level which was independent of mRNA level changes, we speculated that JQ1 elevate FBP1 expression through regulating FBP1 post-translational modifications (PTM). Ubiquitination is one of important post-translational modifications described previously [26]. We found that JQ1 prolonged the half-life of FBP1 protein in a dose dependent manner in PANC-1 cells (Fig. 2i and j). Moreover, JQ1 inhibited FBP1polyubiquitination in dose- and time- dependent manners in PANC-1 cells (Fig. 2k and l). Collectively, these data indicate that JQ1 is capable of inhibiting polyubiquitination and degradation of FBP1 in PDAC.

### JQ1 increases FBP1 protein level through disrupting the interaction between FBP1 and TRIM28

**Fig. 3:**
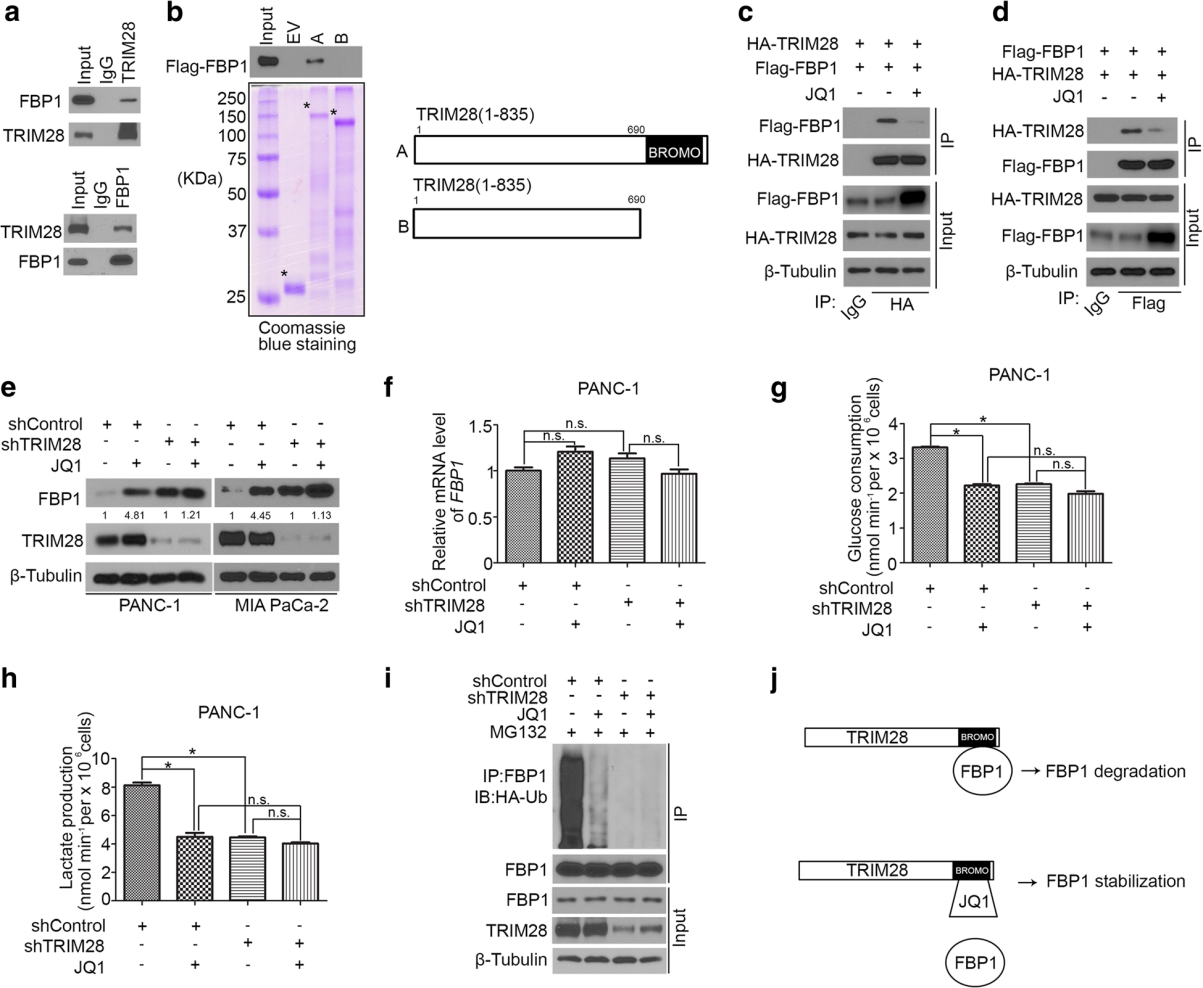
JQ1 increases FBP1 protein level through disrupting the interaction between FBP1 and TRIM28. **a,** reciprocal co-immunoprecipitation of endogenous FBP1 and TRIM28 proteins in PANC-1 cells. **b**, western blot analysis of FBP1 proteins in PANC-1 whole-cell lysate pulled down by GST or GST-TRIM28 recombinant proteins. Schematic diagram depicting a set of GST-TRIM28 recombinant protein constructs. **c and d**, PANC-1 cells were transfected with indicated plasmids. After 24 h transfection, cells were treated with or without 2 μM of JQ1 for another 24 h. Western blot analysis of whole cell lysate and co-IP samples from PANC-1 cells. **e-h**, PANC-1 and SW1990 cells were infected with control or TRIM28-specific shRNAs. After 24 h, cells were treated with or without 2 μM of JQ1 for another 24 h. Cells were harvested for western blot analysis (**e**) and RT-qPCR (**f**) after 48 h transfection. The spent medium was collected for measurement of glucose consumption (**g**) and L-lactate production (**h**). Data are shown as means ± SD (*n* = 3). *, *P* < 0.05; n.s., not significant. **i**, PANC-1 cells were infected with control or TRIM28-specific shRNAs. After 24 h, cells were treated with or without 2 μM of JQ1 for another 24 h. Cells were harvested for western blot analysis. Cells were treated with or without 20 μM of MG132 for 8 h before harvested. **j**, Schematic diagram shows that JQ1 binds to the BOMO domain of TRIM28 and disrupts the interaction of FBP1 with TRIM28 to protect the FBP1 from degradation by TRIM28 in PDAC

It has been reported previously that TRIM28 functions as an E3 ligase of FBP1 in Hepatocellular carcinoma [27]. Indeed, we confirmed the interaction of endogenous FBP1 and TRIM28 in PDAC cell lines (Fig. 3a) and found that the BROMO domain of TRIM28 mediated the binding with FBP1 (Fig. 3b). Due to JQ1 is one of the BET domain inhibitors, we want to investigate whether BET inhibitors stabilize FBP1 through TRIM28. We found that the interaction between FBP1 and TRIM28 was disrupted when JQ1 was added to the immunoprecipitation reaction (Fig. 3c and d). Moreover, we showed that JQ1 could not cause any obvious effect on FBP1 expression in PDAC cell lines after the knockdown of TRIM28 (Fig. 3e and f). Consistent with the above finding, glucose consumption and lactate production were also decreased after JQ1 treatment and this process was blocked by the knockdown of TRIM28 (Fig. 3g and h). Furthermore, we found that JQ1 could no longer reduce FBP1 polyubiquitination after TRIM28 knockdown in PDAC (Fig. 3i). Together, these data suggest that BET inhibitors increase FBP1 protein level through disrupting the interaction between FBP1 and TRIM28 (Fig. 3j).

### FBP1 negatively regulates c-Myc in PDAC

**Fig. 4:**
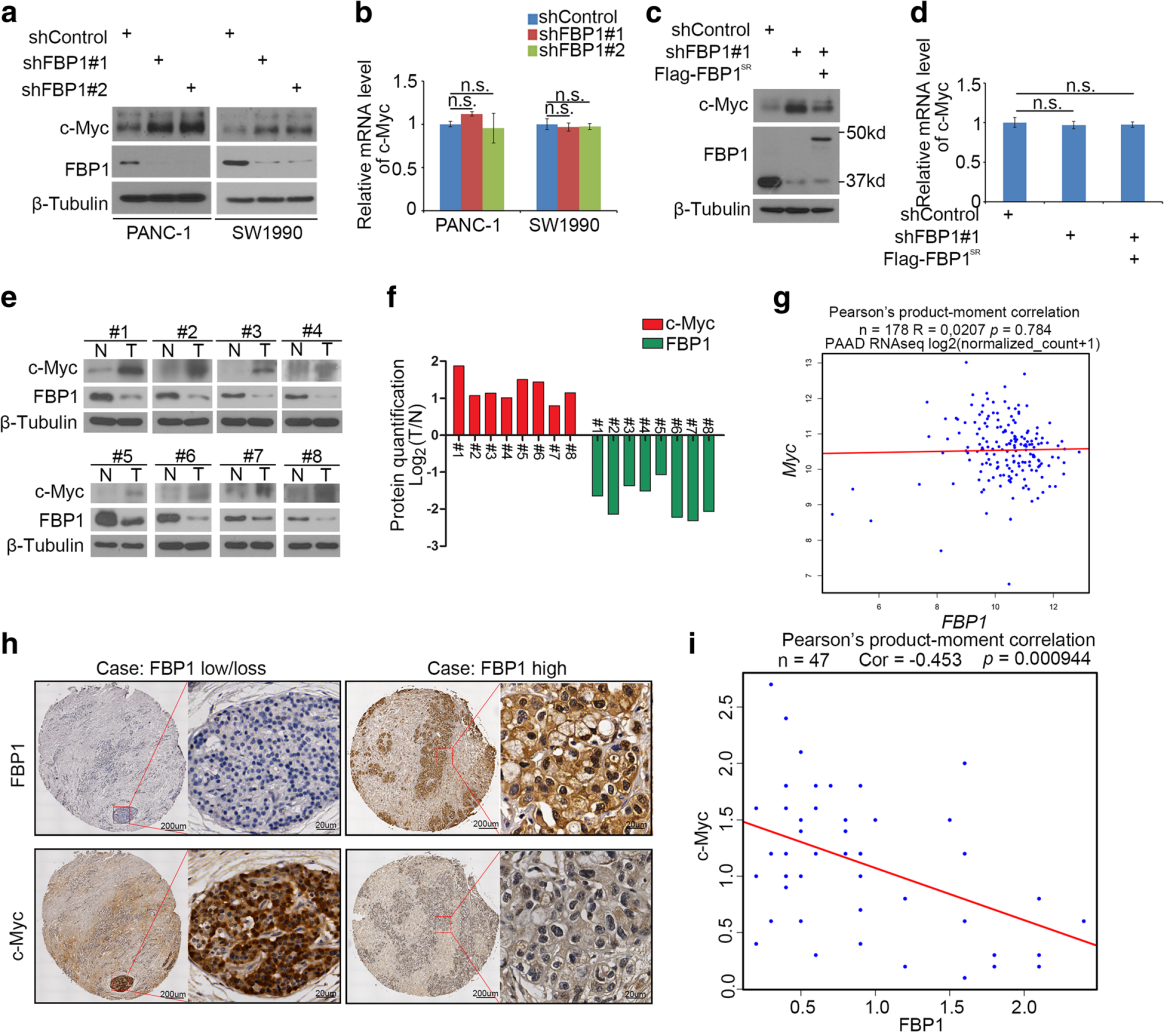
FBP1 negatively regulates c-Myc in PDAC. **a** and **b,** PANC-1 and SW1990 cells were infected with lentivirus expressing control, FBP1-specific shRNAs. 72 h after infection, cells were harvested for western blot analysis (**a**) and RT-qPCR (**b**). Data are shown as means ± SD (n = 3). n.s., not significant. **c-d**, PANC-1 cells cells were infected with lentivirus expressing control, FBP1-specific shRNAs. 48 h after infection, cells were transfected with indicated plasmids. 24 h post transfection, cells were harvested for western blot analysis (**c**) and RT-qPCR (**d**). Data are shown as means ± SD (n = 3). n.s., not significant. **e**, expression of c-Myc and FBP1, as determined by Western blot, in eight paired primary pancreatic cancer tissues (T) and the matched adjacent non-tumor tissues (N) from the same patient. β -Tubulin served as a loading control. **f**, c-Myc and FBP1 proteins were quantified and normalized to the quantified value of β-Tubulin. The quantified value of c-Myc and FBP1 in cancer tissues versus normal tissues was shown in Log_2_ (T/N). **g**, The correlation analysis of mRNA expression levels between FBP1 and Myc was analyzed from TCGA dataset including 178 pancreatic ductal adenocarcinoma. *p* = 0.784, *R* = 0.0207. **h**, images of IHC analysis of FBP1 and c-Myc protein expression on TMA (*n* = 50) tissue sections. Scale bars are shown as indicated. **i**, correlation analysis of the immunoreactivity score of expression levels of FBP1 and c-Myc proteins in human PDAC specimens (n = 50). Pearson’s product-moment correlation co-efficiency and the *p* values are also shown

It has been reported that c-Myc mediates the resistance of pancreatic cancer cells to the BET bromodomain inhibitor JQ1 [9, 10], however the underlying mechanism is under investigation. Therefore, we want to determine whether FBP1 participates in modulating the expression of c-Myc in pancreatic cancer. Firstly, we found that the knockdown of FBP1 increased c-Myc protein level but not the mRNA level in PANC-1 and SW1990 cells (Fig. 4a and b). Then, the restoration of FBP1 after its knockdown decreased c-Myc protein levels but not the mRNA level in PANC-1 cells (Fig. 4c and d). These finding suggest that FBP1 negatively regulates c-Myc protein levels in pancreatic cancer cells.

To investigate the clinical relevance of FBP1 in regulating c-Myc protein levels in pancreatic cancer patients, we assessed both c-Myc and FBP1 protein levels in 8 non-tumor and tumor-paired human pancreatic cancer specimens (Fig. 4e). We found that c-Myc expression was up-regulated in pancreatic cancer tissues compared with adjacent normal pancreatic tissue (Fig. 4e and f). In contrast, the protein level of FBP1 was lower in pancreatic cancer tissues compared to adjacent normal control tissues (Fig. 4e and F). Intriguingly, there was no correlation between *FBP1* and *Myc* at the mRNA level (Fig. 4g). Meanwhile, we used a tissue microarray of human pancreatic cancer specimens obtained from a cohort of patients (*n* = 47 PDAC tissue specimens) [18] to further examine the correlation between FBP1 and c-Myc at the protein level by IHC (Fig. 4h). Statistical analysis of IHC staining revealed that the c-Myc level was inversely correlated with FBP1 in this cohort (Pearson’s product-moment correlation *r* = − 0.453, *p* = 0.000994) (Fig. 4i). Thus, these data suggest that c-Myc is inversely correlated with FBP1 at the protein level in PDAC patient specimens.

### FBP1 promotes c-Myc degradation in pancreatic cancer cells

**Fig. 5:**
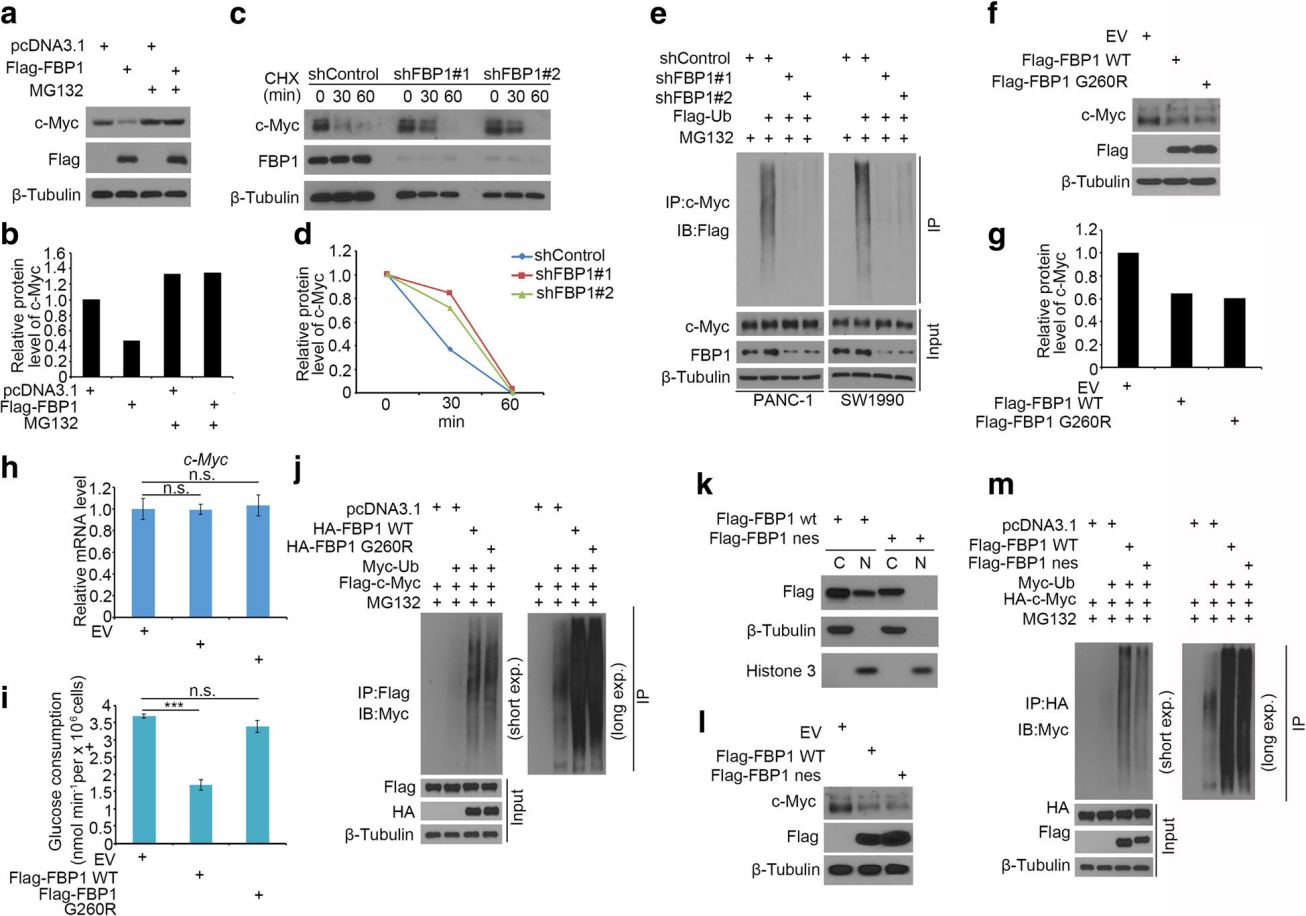
FBP1 promotes c-Myc degradation in pancreatic cancer cells. **a** and **b**, western blot analysis of whole cell lysate of PANC-1 cells transfected with the indicated constructs. Cells were treated with or without 20 μM of MG132 for 8 h before harvest (**a**). The c-Myc protein was quantified and normalized to the quantified value of β-Tubulin (**b**). **c** and **d**, PANC-1 cells were infected with indicated constructs. After 72 h, cells were treated with 50 μg/μl cycloheximide (CHX). At different time points, cells were harvested for western blot analysis. At each time point, the intensity of FBP1 was normalized to the intensity of β-Tubulin (loading control) first and then to the value at the 0-h time point. **e**, PANC-1 and SW1990 cells infected with indicated constructs. Western blot analysis of whole cell lysate of PANC-1 and SW1990 cells after 72 h infection. Cells were treated with or without 20 μM of MG132 for 8 h before harvest. **f-i**, PANC-1 cells were transfected with indicated constructs. 24 h post transfection, cells were harvested for western blot analysis (**f** and **g**), RT-qPCR (**h**) and glucose consumption measurement (**i**). Data are shown as means ± SD (*n* = 3). n.s., not significant; ***, *P* < 0.001. **j**, western blot analysis of whole cell lysate of PANC-1 cells transfected with indicated constructs. Cells were treated with or without 20 μM of MG132 for 8 h before harvest. **k**, western blot analysis of whole cell lysate of PANC-1 cells transfected with indicated constructs. **l**, western blot analysis of whole cell lysate of PANC-1 cells transfected with indicated constructs. **m**, western blot analysis of whole cell lysate of PANC-1 cells transfected with indicated constructs. Cells were treated with or without 20 μM of MG132 for 8 h before harvest

It was noteworthy that FBP1 regulated c-Myc protein levels but not its mRNA level in pancreatic cancer cells (Fig. 4a-d). Consistent with this finding in cultured cells, our clinical data also demonstrated that the FBP1 protein level was inversely correlated with the c-Myc protein level (Fig. 4e), while the *FBP1* mRNA level was not overtly correlated with that of *Myc* (Fig. 4g). Our data suggested that FBP1 regulated c-Myc expression by influencing its post-translational modifications. We systematically investigated whether FBP1 regulate the stability of c-Myc protein in PDAC cells. The overexpression of Flag-FBP1 decreased the protein level of c-Myc in PANC-1 cells, and this effect of FBP1 was completely blocked by the treatment of the proteasome inhibitor MG132 (Fig. 5a and b). Moreover, the knockdown of endogenous FBP1 prolonged the endogenous c-Myc protein half-life in PANC-1 cells (Fig. 5c and d). In agreement with these findings, the knockdown of FBP1 attenuated c-Myc polyubiquitination (Fig. 5e). Therefore, our data indicate that FBP1 promotes the polyubiquitination and degradation of FBP1.

FBP1 is a key enzyme in gluconeogenesis and antagonizes the process of glycolysis [15]. To test whether the enzymatic activity of FBP1 is required for regulating the degradation of c-Myc, we overexpressed the catalytically inactive mutant FBP1 (G260R) [15], and observed a similar inhibitory effect on c-Myc protein level compared with the FBP1 WT in PANC-1 cells (Fig. 5f-5j). These data suggest that FBP1 promotes c-Myc degradation independent of its enzymatic activity. Furthermore, we constructed a mutant FBP1 nuclear export sequence (nes) linked to the C-terminus of FBP1 [15] (Fig. 5k). The overexpression of FBP1 nes had a similar impact as the wild-type FBP1 on c-Myc stability (Fig. 5l and m). These data suggest that FBP1 promotes c-Myc degradation primarily in the cytoplasm.

### FBP1 downregulates c-Myc through inhibition of the ERK1/2 pathway in PDAC

**Fig. 6:**
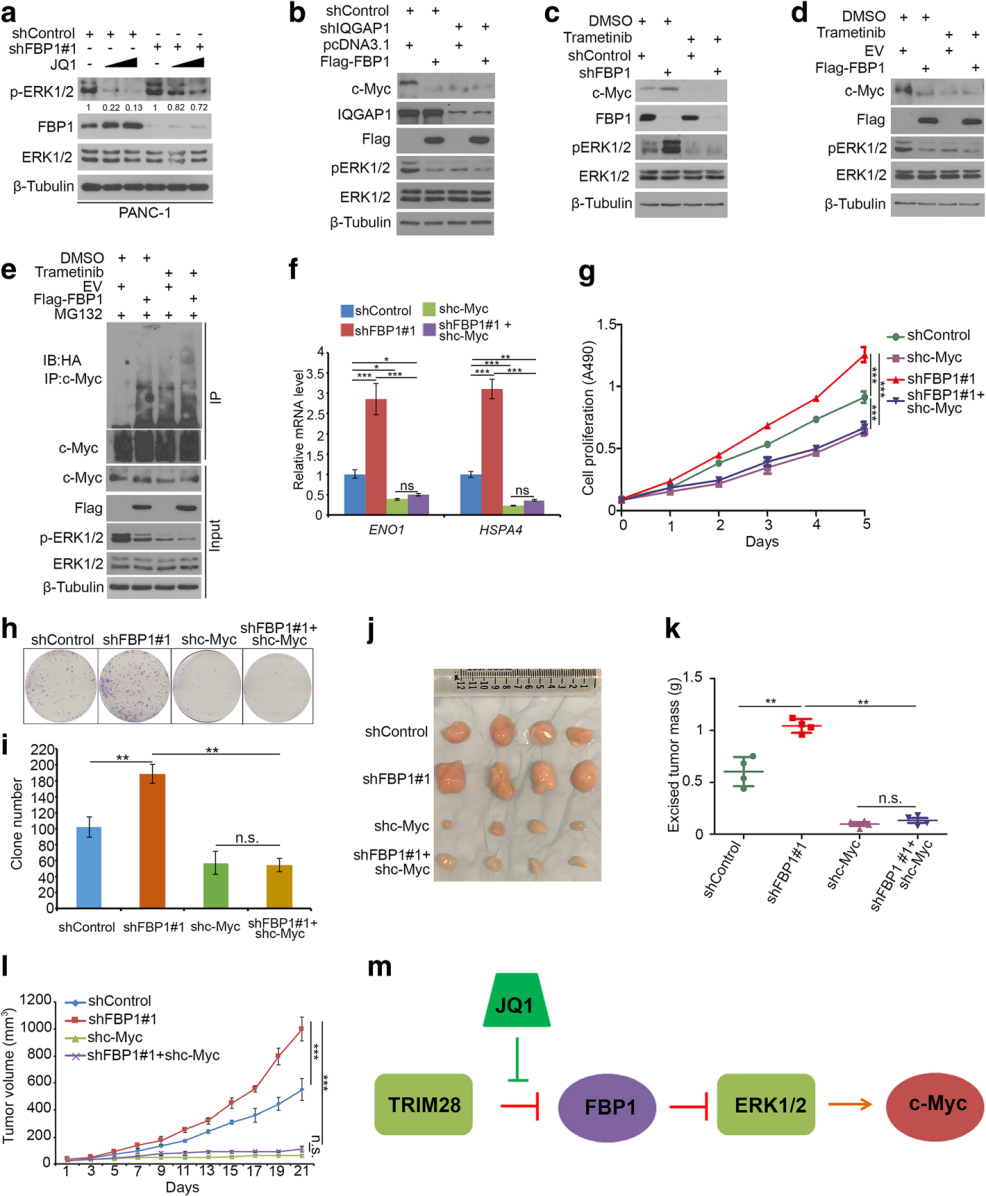
FBP1 downregulates c-Myc through inhibition of the ERK1/2 pathway in PDAC. **a,** PANC-1 cells were infected with control or FBP1-specific shRNAs. After 24 h, cells were treated with or without 2 μM of JQ1 for another 24 h. Cells were harvested for western blot analysis. **b**, PANC-1 cells were infected with lentivirus expressing control, IQGAP1-specific shRNAs. After 48 h infection, cells were transfected with indicated constructs and harvested for western blot analysis 24 h post transfection. Cells were treated with or without 20 μM of MG132 for 8 h before being harvested. **c**, PANC-1 cells were infected with lentivirus expressing control, IQGAP1-specific shRNAs. After 48 h infection, cells were treated with 10 μM of Trametinib for 24 h and harvested for western blot analysis. **d**, PANC-1 cells were transfected with indicated plasmids for 24 h and treated with 10 μM of Trametinib for another 24 h, and harvested for western blot analysis. **e**, PANC-1 cells were transfected with indicared plasmids. 24 h post transfection, cells were treated with 10 μM of Trametinib for 24 h. Cells were harvested for western blot analysis. Cells were treated with 20 μM of MG132 for 8 h before harvest. **f-i,** PANC-1 cells were infected with lentivirus expressing control, or c-Myc and/or FBP1-specific shRNAs. After 48 h infection and puromycin selection, cells were harvested for RT-qPCR analysis (**f**), MTS assay (**g**) and colony formation assay (**h** and **i**) after 48 h infection. For rt-qPCR, all data show mean values ± SD (error bar) (n = 3); for MTS assay, data are shown as means ± SD (*n* = 6); for colony formation assay, data are shown as means ± SD (n = 3). n.s., not significant; * *P* < 0.05; ** *P* < 0.01; ***P < 0.001. **j-l**, After 72 h infection, cells were injected into subcutaneous layer of the right flank of mice. The growth of tumor was measured every other day for 21 days (**l**), and tumors were harvested and measured the weight of tumor (**k**). Data present means ± SD (*n* = 4). n.s., not significant; **P < 0.01; ***P < 0.001. (**m**) A hypothetical model depicting JQ1 disrupts the interaction between FBP1 and TRIM28 and stabilizes FBP1.Next, FBP1 promoted c-Myc degradation through inhibiting the activation of ERK1/2. FBP1 serves as a therapeutic niche for patient-tailored therapies

It has previously been reported that FBP1 inhibits the IQGAP1–ERK interaction and decreases the phosphorylation of ERK1/2 in pancreatic cancer [17]. Firstly, we demonstrated that JQ1 could decrease ERK1/2 phosphorylation (p-ERK1/2) and this process was attenuated by knockdown of FBP1 in PANC-1 cells (Fig. 6a), indicating that BET inhibitors regulate ERK activation through FBP1. Furthermore, 90% of PDAC patients were found to harbor Ras mutants [28]. Ras signaling and effector extracellular signal-regulated kinase (ERK) activation lead to the stabilization of c-Myc by attenuating its ubiquitin-mediated protein degradation [29, 30]. We sought to determine whether FBP1 destabilize c-Myc in an IQGAP1-ERK pathway-dependent manner. The knockdown of IQGAP1 not only decreased the phosphorylation of ERK and c-Myc protein levels but also blocked the effect of FBP1 on the downregulation of c-Myc in PANC-1 cells (Fig. 6b). Moreover, we treated PANC-1 cells with a MEK inhibitor (trametinib) to block ERK phosphorylation (Fig. 6c and d). In agreement with previous findings, the MEK inhibitor decreased c-Myc levels and ERK phosphorylation [31]. In addition, we found that the FBP1 knockdown (Fig. 6c) or the ectopic expression of FBP1 (Fig. 6d and e) failed to affect the expression of c-Myc in ERK-inactivated cells. Thus, our data suggest that FBP1 promotes c-Myc degradation through the inhibition of the ERK pathway in pancreatic cancer cells, and the BET inhibitor downregulates c-Myc partially via stabilizing FBP1 in pancreatic cancer cells.

Previous studies have shown that FBP1 is downregulated in many types of solid tumors and that the restoration of FBP1 inhibits tumor cell proliferation [17]. Since our data showed that FBP1 promoted c-Myc degradation, we want to investigate the role of c-Myc on FBP1-mediated tumor suppression. We infected PANC-1 cells with the control, FBP1- and/or c-Myc-specific shRNA and harvested the lentivirus-infected cells for RT-qPCR, MTS assay, colony formation and animal studies. The knockdown of FBP1 increased the mRNA expression of c-Myc downstream target genes (*HSPA4* and *ENO1*) [32], and this process was impeded by the knockdown of c-Myc in PANC-1 cells (Fig. 6f). MTS and colony formation assays showed that the knockdown of FBP1 increased cell growth, while the co-knockdown of c-Myc attenuated FBP1 knockdown-induced cell proliferation (Fig. 6g-h). Furthermore, the knockdown of FBP1 alone promoted the tumor growth in mice, but the knockdown of c-Myc alone inhibited this growth (Fig. 6j-6l). The co-knockdown of FBP1 and c-Myc blocked FBP1 knockdown-induced tumor growth in mice (Fig. 6k-6l). These data suggest that FBP1 decreases pancreatic cancer cell growth via inhibition of ERK-c-Myc pathway.

## Discussion

FBP1 is a rate-limiting enzyme in gluconeogenesis that antagonizes glycolysis by converting fructose-1, 6-bisphosphate to fructose-6-phosphate and inorganic phosphate [27]. It is considered a tumor suppressor gene due to its role in the inhibition of aerobic glycolysis and subsequently impeding cancer cell proliferation [15]. Indeed, the downregulation of FBP1 leads to tumor progression and poor prognosis in pancreatic cancer [17], and this is also the case in various types of tumor [27, 33, 34]. Therefore, an up-regulation of FBP1 in pancreatic cancer might provide an ideal strategy to inhibit the pancreatic cancer cell progression. The specific mechanism underlying FBP1 downregulation is associated with promoter methylation and copy-number loss, leading to decreased FBP1 mRNA expression [22, 35] or post-transcriptional modification of FBP1 protein mediated by MAGE-TRIM28 complex, which results in FBP1 degradation in cancer cells [27]. Our findings in this study suggest that JQ1 increases FBP1 stability through disrupting the interaction between FBP1 and TRIM28, which suggest JQ1 might be an ideal small molecular to increase the FBP1 expression and repress pancreatic cancer.

A number of bromodomain inhibitors are currently being tested in several clinical trials [36], making those potentially promising drugs for the treatment of pancreatic cancer. BET inhibitor resistance often emerges in various cancer types [3, 37, 38]. Many factors, such as increasing the BRD4 protein level [24, 39, 40], were proved to lead drug resistance to BET inhibitors. Our data identified that FBP1 was a novel factor influencing the sensitivity of BET inhibitors. Due to c-Myc mediating the resistance of pancreatic cancer cells to the BET bromodomain inhibitor JQ1 [9, 10], our data also show that FBP1 decreases the expression of c-Myc in pancreatic cancer, which provides a reasonable mechanism to explain how FBP1 sensitized BET inhibitors treatment in pancreatic cancer.

Approximately 90% of PDAC patients possess Ras mutations [28]. It has been reported that Ras signaling and effector extracellular signal-regulated kinase (ERK) activation lead to the stabilization of c-Myc by attenuating its ubiquitin-mediated protein degradation [29, 30]. It is worth noting that FBP1 could bind to the WW domain of IQGAP1 and block IQGAP1-dependent ERK1/2 phosphorylation in pancreatic cancer [17]. In this study, we demonstrate that FBP1 promotes c-Myc degradation by inhibiting the MAPK pathway in pancreatic cancer cells. Intriguingly, it has been well documented that BRD4 directly regulates c-Myc expression at the transcriptional level [41]. In addition, it is reported that JQ1 decreases the mRNA level of c-Myc [42]. While, combined with our previous findings, the BET inhibitor increases FBP1 protein level which promoted c-Myc degradation in pancreatic cancer cells. Therefore, our data suggest that BET inhibitors regulate c-Myc expression not only in the transcription level but also in the post-transcription level.

## Conclusions

FBP1 is responsible for the sensitivity of treatment with BET inhibitors in pancreatic cancer. JQ1 increases FBP1 protein level by disruption the interaction between TRIM28 and FBP1 (Fig. 3j and 6m). Moreover, we show that FBP1 promotes c-Myc degradation through inhibition of the MAPK pathway (Fig. 6m). Together, our data suggest that FBP1 modulates the sensitivity of BET inhibitors by decreasing the expression of c-Myc in pancreatic cancer. These findings highlight FBP1 as a therapeutic niche for patient-tailored therapies.

## Additional file

Additional file 1: **Figure S1.** BRD2, BRD3 or BRD4 make no effect on the expression of FBP1. **Table S2.** Sequences for shRNAs. (ZIP 272 kb)

## Abbreviations

BET: Bromodomain and extraterminal domain
CHX: cycloheximide
co-IP: Co-immunoprecipitation
FBP1: Fructose-1, 6-biphosphatase
GST: Glutathione S-transferase
PDAC: Pancreatic ductal adenocarcinoma
