# Supplementary material for: FBP1 loss contributes to BET inhibitors resistance by undermining c-Myc expression in pancreatic ductal adenocarcinoma
Source: J Exp Clin Cancer Res. 2018 Sep 10;37:224. doi: 10.1186/s13046-018-0888-y (PMC6131902; doi:10.1186/s13046-018-0888-y)
Supplement: Supplementary file 1 — Figure S1. BRD2, BRD3 or BRD4 make no effect on the expression of FBP1. Table S2. Sequences for shRNAs. (ZIP 272 kb) [file 13046_2018_888_MOESM1_ESM.zip › Supplementary information .docx]

**Supplementary Information**

**FBP1 loss contributes to BET inhibitors resistance byundermining c-Myc expression in pancreatic ductal adenocarcinoma**

Bo Wang, Ping Fan, Jingyuan Zhao, Xin Jin and Heshui Wu

**Supplementary Figure Legends**

**Supplementary figure 1. a and b,** PANC-1 cells were infected with lentivirus expressing control, BRD2-specific shRNAs. 72 h after infection, cells were harvested for western blot analysis (a) and RT-qPCR (b). Data are shown as means ± SD (n = 3). n.s., not significant. **c and d**, PANC-1 cells were infected with lentivirus expressing control, BRD3-specific shRNAs. 72 h after infection, cells were harvested for western blot analysis (c) and RT-qPCR (d). Data are shown as means ± SD (n = 3). n.s., not significant. **e and f**, PANC-1 cells were infected with lentivirus expressing control, BRD4-specific shRNAs. 72 h after infection, cells were harvested for western blot analysis (e) and RT-qPCR (f). Data are shown as means ± SD (n = 3). n.s., not significant.

**Supplementary Table S1: Sequences of primers for RT-qPCR**

| **Species** | **Gene** | **Forward (5’-3’)** | **Reverse (5’-3’)** |
| --- | --- | --- | --- |
| Human | *FBP1* | ACATCGATTGCCTTGTGTCC | CCACCAAAATGAACTCCCCG |
| Human | *β-actin* | CCCTGGCTCCTAGCACCAT | AGAGCCACCAATCCACACAGA |
| Human | *Myc* | TTCGGGTAGTGGAAAACCAG | CAGCAGCTCGAATTTCTTCC |
| Human | *HSPA4* | AGCCAAGAAGGCAAAAGTGA | CCACTGCGTTCTTAGCATCA |
| Human | *ENO1* | TGATCGAGATGGATGGAACA | CGCCATTGATGACATTGAAC |

**Supplementary Table S2: Sequences for shRNAs**

| shFBP1-1 | 5′- CCGGCCTTGATGGATCTTCCAACATCTCGAGATGTTGGAAGATCCATCAAGGTTTTTG -3′ |
| --- | --- |
| shFBP1-2 | 5′- CCGGCGACCTGGTTATGAACATGTTCTCGAGAACATGTTCATAACCAGGTCGTTTTTG -3′ |
| shIQGPA1-1 | 5′- CCGGGCCCACATTGTGCCTTTATTTCTCGAGAAATAAAGGCACAATGTGGGCTTTTTG -3′ |
| shIQGAP1-2 | 5′- CCGGCCTCAGATTCAAGACCTATATCTCGAGATATAGGTCTTGAATCTGAGGTTTTTG -3′ |
| shMyc-1 | 5′- CCGGCCTGAGACAGATCAGCAACAACTCGAGTTGTTGCTGATCTGTCTCAGGTTTTTG -3′ |
| shMyc-2 | 5′- CCGGCAGTTGAAACACAAACTTGAACTCGAGTTCAAGTTTGTGTTTCAACTGTTTTTG -3′ |
| shBRD2-1 | 5′-CCGGCCGGAAGCCCTACACCATTAACTCGAGTTAATGGTGTAGGGCTTCCGGTTTTTG -3′ |
| shBRD2-2 | 5′-CCGGCCTACCACTGTCCTCAACATTCTCGAGAATGTTGAGGACAGTGGTAGGTTTTT -3′ |
| shBRD3-1 | 5′-CCGGGTGAGATTCGTACCGAAGAACCTCGAGGTTCTTCGGTACGAATCTCACTTTTTTG-3′ |
| shBRD3-2 | 5′-CCGGCAAATTGAACCTGCCGGATTACTCGAGTAATCCGGCAGGTTCAATTTGTTTTTTG-3′ |
| shBRD4-1 | 5′-CCGGCCTGGAGATGACATAGTCTTACTCGAGTAAGACTATGTCATCTCCAGGTTTTTG-3′ |
| shBRD4-1 | 5′-CCGGCAGTGACAGTTCGACTGATGACTCGAGTCATCAGTCGAACTGTCACTGTTTTTG-3′ |
