# Supplementary figures and images for: FBP1 loss contributes to BET inhibitors resistance by undermining c-Myc expression in pancreatic ductal adenocarcinoma
Source: J Exp Clin Cancer Res. 2018 Sep 10;37:224. doi: 10.1186/s13046-018-0888-y (PMC6131902; doi:10.1186/s13046-018-0888-y)

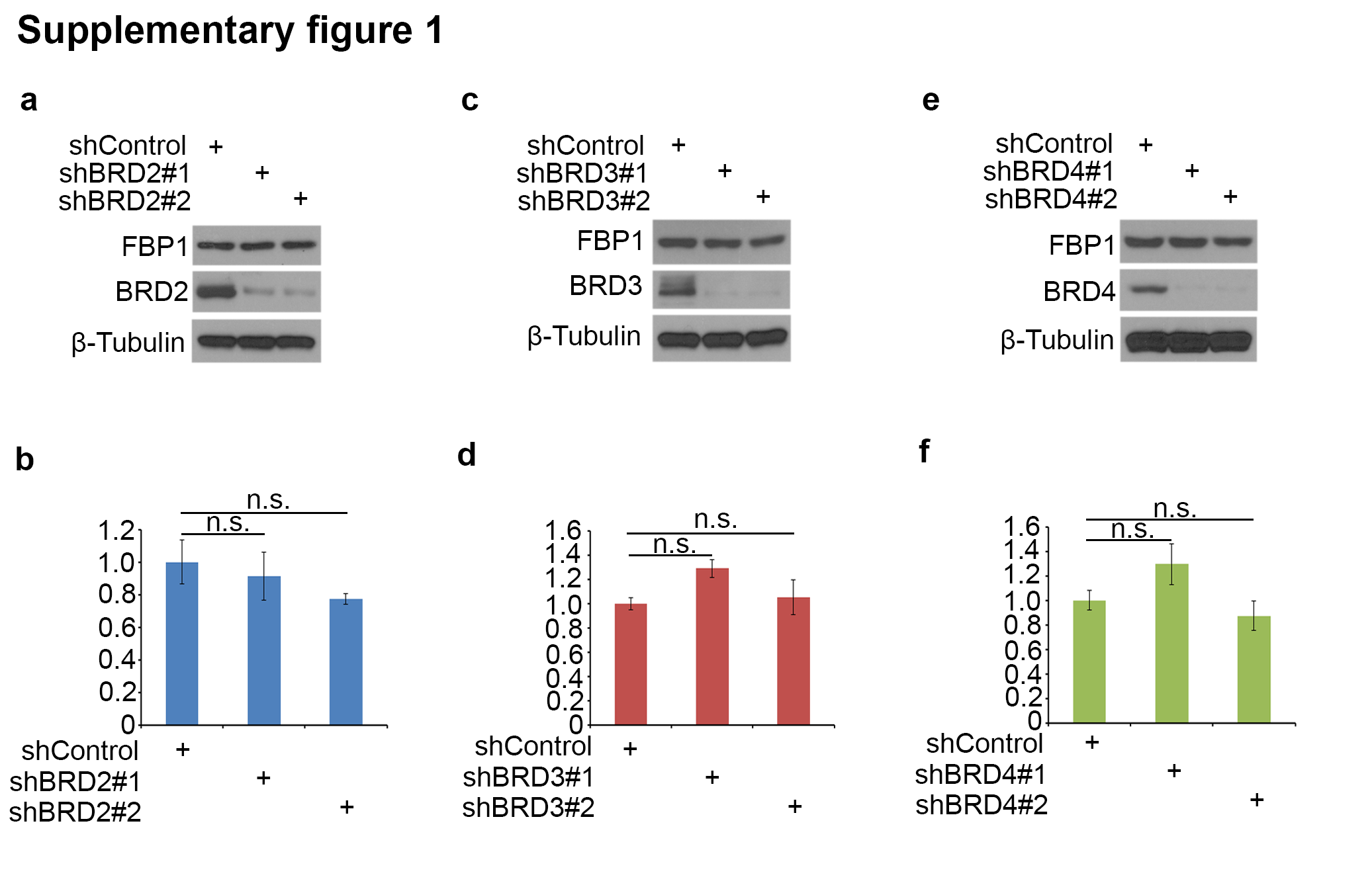

Supplement: Supplementary file 1 — Figure S1. BRD2, BRD3 or BRD4 make no effect on the expression of FBP1. Table S2. Sequences for shRNAs. (ZIP 272 kb) [file 13046_2018_888_MOESM1_ESM.zip › Supplementary figure 1 re.tif]
